# Comparison of the Separate and Combined Effects of Physiotherapy Treatment and Corticosteroid Injection on the Range of Motion and Pain in Nontraumatic Rotator Cuff Tear: A Randomized Controlled Trial

**DOI:** 10.1155/2021/6789453

**Published:** 2021-10-25

**Authors:** Shaahin Hajivandi, Atousa Dachek, Amirhossein Salimi, Hesan Jelodari Mamaghani, Seyed Peyman Mirghaderi, Javad Dehghani, Roham Borazjani, Amirmohammad Babaniamansour, Rojin Sarallah, Salar Javanshir, Maryam Salimi

**Affiliations:** ^1^Department of Orthopedic Surgery, Dezful University of Medical Sciences, Dezful, Iran; ^2^Student Research Committee, Dezful University of Medical Sciences, Dezful, Iran; ^3^Student Research Committee, Shahid Sadoughi University of Medical Sciences, Yazd, Iran; ^4^Students' Scientific Research Center (SSRC), Tehran University of Medical Sciences, Tehran, Iran; ^5^Bone and Joint Diseases Research Center, Department of Orthopedic Surgery, Shiraz University of Medical Sciences, Shiraz, Iran; ^6^Trauma Research Center, Shahid Rajaee (Emtiaz) Ttauma Hospital, Shiraz University of Medical Sciences, Shiraz, Iran; ^7^Department of Orthotics and Prosthetics, School of Rehabilitation Sciences, Iran University of Medical Sciences, Tehran, Iran; ^8^Islamic Azad University Medical Branch of Tehran, Tehran, Iran; ^9^Student Research Committee, Shiraz University of Medical Sciences, Shiraz, Iran

## Abstract

**Background:**

Surgical treatment and conservative treatment is the options to improve pain, function, and range of motion following rotator cuff tear. In this study, we aimed to compare the effects of physiotherapy and corticosteroid injections on the function, pain, and range of motion in patients with full-thickness rotator cuff tearing separately and simultaneously.

**Methods:**

A total of 96 patients were randomly assigned to the study and divided into 3 groups of 32 patients. DASH questionnaire and VAS criterion were completed by all three groups, and the range of motions of all groups was measured by a goniometer. Then, the first group underwent 12 sessions of physiotherapy twice a week for 6 weeks; the second group received 80 mg of methylprednisolone and 1 ml of lidocaine 2% in two stages, 21 days apart; and the third group received 80 mg of methylprednisolone and 1 ml of lidocaine 2%, and after 2 days, 6 sessions of physiotherapy twice a week for 3 weeks were prescribed. In the end, the questionnaire was filled out by the patient, and the range of emotions was assessed with a goniometer.

**Results:**

More than 80% of patients in each group were female. There was no significant difference between the gender and age distribution of the groups. The mean age in physiotherapy, steroid, and physiotherapy + steroid groups was 51.78 ± 7.37, 52.37 ± 6.61, and 50.87 ± 5.65, respectively. The combination of physiotherapy + steroid intervention was more effective in reducing VAS and DASH scores than physiotherapy or steroid injection alone. Goniometric findings showed that treatments that included the steroid injection approach (steroid injection and steroid + physiotherapy) had a more dramatic effect on improving the patients' range of motion than physiotherapy alone.

**Conclusions:**

Among the conservative approaches of treating full-thickness rotator cuff tear, a combination of steroid injection and physiotherapy is more effective significantly in comparison with either treatment alone. This trial is registered with IRCT20200102045987N1.

## 1. Introduction

The shoulder is one of the largest joints and the most mobile and unstable joint in the body due to the shallow depth of the glenoid cavity. Therefore, the tendons and muscles around the joint including the rotator cuffs (e.g., supraspinatus, infraspinatus, teres minor, and subscapularis) play an important role in joint stability as well as joint movements [1]. Furthermore, shoulder pain with muscle-associated origin is one of the most common complaints of patients who visit orthopedists (30% to 70%), among which rotator cuff ruptures are considered as the most prevalent ones (23% to 49%) [2].

Pain, weakness, and limited range of motion are the most common symptoms of complete rotator cuff rupture, which can impair the function and quality of life [3]. Likewise, magnetic resonance imaging (MRI) was recognized as a gold standard diagnostic test regarding rotator cuff tearing [4].

The prevalence and size of the ruptures increase with age, and the clinical success of the tendon repair decreases within the ruptures [5]. In this regard, treatment is performed in conservative or surgical management including open surgery and arthroscopy. Based on the patient's condition and the rapture size as well as available equipment and facilities, the type of treatment is selected; in fact, not all patients with rotator cuff ruptures need surgery. Moreover, the treatment choice for rotator cuff tearing was varied in the studies with different lengths of the follow-up period and complications [6].

Although many studies have reported the results of complete rotator cuff rupture surgery, there are few studies on the effectiveness of conservative treatment. Likewise, conservative and physiotherapy treatments are diverse and choosing the best and most appropriate treatment for these patients requires further research.

Therefore, due to the high prevalence and functional disability caused by rotator cuff tearing, disagreement about the appropriate treatment method and the limited evidence regarding the role of physiotherapy and exercise programs in reducing pain and disability of these patients, and lack of sufficient objective evidence of complete recovery of patients following surgery, a randomized clinical trial was needed to reduce the complications, pain, and costs of surgical treatment and determine the effects of physiotherapy and corticosteroid injections (CSI) in these patients.

Numerous studies have been performed in recent years on the effects of physiotherapy and CSI in patients with other pathologies of the rotator cuff muscles such as impingement syndrome and frozen shoulder, which are pathologically different from the complete tendon rupture. In the present study, the separate and combined effect of physiotherapy and CSI on motor function and pain in patients with complete rupture of the rotator cuff was investigated.

## 2. Materials and Methods

In the present randomized clinical trial study, 96 patients (15 males and 81 females) with a mean age of 51 years who had complete rotator cuff rupture in the period of February 2017 to November 2016 were identified and enrolled in the study.

According to the specific purpose of comparing the performance in the three groups under study, the opinion of the relevant experts and previous studies [7] and considering *p*1=0.63 (percentage of the effect of physiotherapy and CSI on motor function in patients with rotator cuff rupture) and *p*2=0.3 (percentage of the effect of physiotherapy on motor function of patients with rupture of the rotator cuff), the sample size was calculated using the following formula in the three intervention groups.

(1) $$\begin{array}{c} n=\frac{{\left(z_{1-\left(a/2\right)}+z_{1-\beta }\right)}^{2}\left[p^{1}\left(1-p^{1}\right)+p^{2\left(1-p^{2}\right)}\right]}{{\left(p^{1}-p^{2}\right)}^{2}},\\ n=\frac{\left(1/96+0.85\right)2\;\left[0/63\left(1-0.3\right)\;+\;0.3\left(1-0.7\;\right)\right]}{\left(0/63-0/3\right)2}=32. \end{array}$$

Therefore, the first group (physiotherapy and CSI) of 32 people, the second group (physiotherapy) of 32, and the third group (CSI) of 32 were calculated.

Inclusion criteria were patients with an age range of 30 to 70 years who were diagnosed with complete rotator cuff degenerative rupture with MRI and clinical examination and were consciously and cooperatively willing to participate in the study. Exclusion criteria were any concomitant shoulder disease such as infection, fracture, and tumor as well as trauma during treatment and therapeutic intervention by other people. Patients were referred to the orthopedic clinic and assessed after signing the informed consent. The variables studied were pain and active range of motion of patients. The severity of pain was evaluated based on the visual pain scale (VAS), and the active range of motion was assessed by goniometer and disabilities of the arm, shoulder, and hand (DASH) questionnaire.

The DASH questionnaire has 30 questions, of which 23 are about functional limitations in work, play, and interaction and examine the inadequacy of the upper extremity in performing functional activities, and 7 questions are about the severity of symptoms such as pain and weakness in impairing a person's functional ability. Likewise, the questionnaire is scored using a Likert scale with a range of 1 to 5 for each question. Score 1 indicates the absence of difficulty or limitation in the activities in question, and score 5 indicates the high intensity of the constraint. According to the instructions to convert the scores resulting from the questionnaire to standard scores in the range of 0 to 100 with the standard deviation of 50 ± 10, the raw scores obtained from each questionnaire are included in the following formula:

(2) $$\begin{array}{c} \left(\frac{\text{total answers to questions}}{\text{number of answered questions}}-1\right)\times 25. \end{array}$$

Therefore, the overall score of this questionnaire is between zero and 100, and the higher scores indicate a higher level of disability. [8] Furthermore, it has shown that the DASH questionnaire is well able to distinguish between different levels of functional disability [9]; consequently, it is inferred that scores close to 100 in the DASH questionnaire represent higher levels of functional disability and inadequacy in comparison with the scores close to zero, which are considered as functional adequacy, and in the same way, scores close to 50 are in the intermediate range of this spectrum.

In addition to the 30 questions in this questionnaire, there are two series of questions with four items that are optional to answer and are called sports/arts DASH and work DASH and are scored similarly above. This part is not used in the present study. Mousavi et al. [10] prepared a Persian version of this tool and standardized the process in 271 Iranian patients with musculoskeletal disorders of the upper extremities such as painful arch syndrome, carpal tunnel syndrome, joint capsule adhesions, ulnar nerve entrapments, tendonitis, and bursitis. Cronbach's alpha was 0.96, and the intraclass correlation coefficient was 0.82. The present study uses the validated Persian version of this questionnaire.

Visual pain scale (VAS) is the pain ruler that contains a horizontal line that is scaled from zero to 10; zero indicates absolutely no pain, and 10 signs the unbearable and worst pain [11].

After the initial evaluation, the patients were randomly divided into 3 groups of 32 patients with block static method, so that based on 9-person blocks (AABBCC), all its subitems were calculated and randomly assigned to each intervention group.

The first group underwent 12 sessions of physiotherapy in the form of massage, rotator cuff and scapular muscle stretching exercises, strengthening exercises and modalities including laser, transcutaneous electrical nerve stimulation (TENS), and ultrasound (US) twice a week for 6 weeks with a duration of 45 minutes per session including 30 minutes of electrotherapy and 15 minutes of manual therapy training. Normal tense current with a frequency of 120 Hertz, a pulse width of 120 microseconds in 15 minutes and current with a frequency of 50 Hertz, a pulse width of 350 microseconds, an 8 second contraction time, and a 2 second rest time for 10 minutes were used. Ultrasound with 1 to 2 watts per frequency of 3 MHz continuously and intensity of 5 cm square in 5 minutes was performed by a specialized rehabilitation physiotherapist. The first stage included the first 4 sessions to achieve shoulder movements, especially internal and external rotation equal to the opposite side and arm abduction less than 90°; with Cadman pendulum exercises and passive range of motion exercises, bending the shoulder, straightening the shoulder, and internal and external rotation were started. Capsule stretching exercises for the anterior, posterior, and inferior capsules were taught to patients using the contralateral arm and gradually moved toward active range-of-motion exercises such as wall walking. All inactive exercises at this stage were performed up to the maximum range of motion that did not aggravate the patient's pain, with the aim of controlling pain and creating shoulder movements below 90° and elbow movements.

The second phase consisted of 4 second sessions performed with the aim of improving the strength, power, and endurance of the shoulder set. Pain control methods such as the first step were also performed in this step. The goal of the shoulder movements at this stage was to equalize the active range of motion of the affected shoulder joint with the opposite shoulder in all movement plates. For this purpose, exercises included passive range of motion, capsular stretching, and auxiliary-active movement exercises; then, the range-of-motion exercises were performed. To gain muscle strength, we performed the exercises twice a week, with 8 to 12 repetitions for three periods. Exercises included strengthening the remaining rotator cuff muscles, starting closed-chain isometric reinforcement (internal and external rotation and abduction), and progressing to open-chain strengthening.

The third step was performed in the third 4 sessions with the aim of improving neuromuscular control and shoulder depth sense, preparing for a gradual return to functional activities. At the end of the sessions, motor function and pain according to DASH and VAS questionnaires and measurements with a goniometer (flexion, extension, abduction, and external and internal rotation) were evaluated.

The second group was injected with 8 mg of methylprednisolone and 1 ml of 2% lidocaine with a posterior approach in the subacromial region in two stages 21 days apart. Two weeks after the second injection, the patient completed a questionnaire, and functional assessments were examined with a goniometer.

The third group was injected with 8 mg of methylprednisolone and 1 ml of lidocaine 2% and, two days later, underwent 6 sessions of physiotherapy by a rehabilitation specialist twice a week for 3 weeks. The duration of each session was 45 minutes, including 30 minutes of electrotherapy and 15 minutes of manual therapy training; after 6 sessions of physiotherapy at the end of the third week, the injection was performed, and the other 6 sessions of physiotherapy twice a week for 2 weeks were performed with a duration of 45 minutes per session. (The steps of physiotherapy were the same as the first group.)

All groups were instructed not to use NSAIDs or herbal and home remedies either orally or systemically or topically during the study. At the end of the sessions, function and pain were assessed according to the DASH and VAS questionnaires and goniometer measurement. At the end of the study, the data before and after the intervention of all three groups were compared.

Ethical considerations were applied in accordance with the approval of the ethics committee of Dezful University of Medical Sciences with the code number IR.DUMS.REC.1398.045. All the patients who agreed to participate in the trial provided written informed consent. Likewise, the patients' personal information was also preserved. The study protocol, including the statistical analysis plan, is available at fa.irct.ir with the code number IRCT20200102045987N1. Quality control of verification and screening of adherence to protocols were performed on a regular basis by the trial coordinators. This research has been approved by the IRB of the authors' affiliated institutions. One-way analysis of variance within the subjects and Friedman test were used to investigate the relationship between separate and combined physiotherapy treatment and CSI and the studied variables. Statistical analysis was performed using SPSS software version 23. Alpha less than 0.05 was considered significant.

## 3. Results

In this study, 32 patients in each treatment group were present. More than 80% of the patients in each group were female. The chi-square test shows that there is no significant difference between the gender distribution of groups (*p*=0.789). The gender frequency distribution of patients in different groups was summarized in Table 1. The mean age in the physiotherapy, steroid, and physiotherapy + steroid groups was 51.75 ± 7.37, 52.37 ± 6.61, 50.87 ± 5.65 years, respectively. ANOVA test showed that there was no significant difference in the age of the three groups (*p*=0.658).

Paired samples *T*-test shows that in all three groups, the DASH and VAS scores have decreased significantly after the intervention. In other words, all three interventions have been effective in reducing the DASH and VAS scores. Also, the ANOVA test showed that there was no difference in DASH and VAS scores between patients before the intervention, but after the intervention, Bonferroni and Scheffe post hoc test showed that DASH and VAS scores were not different in the physiotherapy and steroid groups, but the DASH as well as VAS score in the physiotherapy + steroid group was significantly lower than the other two groups. In other words, the combined physiotherapy + steroid intervention was more effective in reducing the DASH and VAS scores (*p* < 0.05; Table 2).

Paired samples *T*-test shows that in all three groups, after the intervention, the flexion angle increased significantly. In other words, all three interventions were effective in increasing the flexion angle. The ANOVA test showed that there was no difference in the patients' flexion angle before the intervention, but this difference was significant after the intervention. Bonferroni and Scheffe post hoc tests showed that the flexion angle in the steroid group and physiotherapy + steroids were not different, but the angle of flexion in the physiotherapy group was significantly less than the other two groups. In other words, physiotherapy was less effective in increasing the angle of flexion than treatments containing steroids (*p* < 0.05; Table 2).

After intervention in the steroid and physiotherapy + steroid groups, the extension angle increased significantly (*p* value = 0 and 0.029, respectively), but this increase was little. The ANOVA test also showed that there was no difference in the extension angle of patients before and after the intervention. Therefore, the interventions performed did not change the extension angle clinically and significantly (Table 2).

In all three groups, the abduction angle increased significantly after the intervention. In other words, all three interventions were effective in increasing the abduction angle. Also, the ANOVA test showed that there was no difference in the abduction angle of patients before the intervention, but after the intervention, this difference was significant. Bonferroni and Scheffe post hoc test showed that the abduction angle in the physiotherapy + steroid group was more than in the other two groups, and in the steroid group, it was more than the physiotherapy group. In other words, physiotherapy + steroid was the most effective treatment in increasing the abduction angle, and physiotherapy alone was less effective (*p* < 0.05; Table 2).

Paired samples *T*-test showed that in all three groups, after the intervention, the internal and external rotation angles increased significantly. In other words, all three interventions were effective in increasing the internal and external rotation angles. ANOVA test also showed that there was no difference in the patients' internal and external rotation angles before the intervention, but this difference was significant after the intervention. Bonferroni and Scheffe post hoc test showed that there was no significant difference in the internal and external rotation angles in the steroid group and physiotherapy + steroid, but this angle in the sole physiotherapy group was significantly less than the other two groups, which received containing steroid therapies (*p* < 0.05).

## 4. Discussion

Management of rotator cuff complete rapture is a dilemma due to the controversial opinion among various surgical and nonsurgical treatment modalities. Nowadays with respect to surgical complications (e.g., stiffness, infection, neurologic injury, bleeding, prolonged hospital stays, and so on), there is an increasing trend for shifting from operative techniques to more conservative, nonoperative, and less invasive methods [12]. Likewise, with respect to fewer side effects and cost as well as more accessibility of conservative treatment, it seems that it is more preferable the first-line treatment in comparison to surgery. Furthermore, Ryösä et al. concluded in a systematic review that the surgical approach did not perform significantly better in terms of pain and function than conservative treatment [13]. Moreover, there are several studies that also suggested conservative therapy as the first-line treatment of degenerative rotator cuff tears except in those with acute traumatic rapture [14–17]. On the other hand, the American Academy Orthopaedic Surgeons (AAOS) guidelines claimed that surgical repair is a superior valid option for patients with symptomatic full-thickness rotator cuff tears even though there is also a lack of supporting evidence for conservative treatment [18]. Moreover, the superiority of surgical over the conservative treatment is challenging to demonstrate, due to the heterogeneity of studies' findings. According to the recent meta-analysis, significant differences were reported in terms of the Constant–Murley score and VAS only at 1 year follow-up in favor of the surgical group compared with the conservative group [12]. On the other study, the short time follow-up of both the surgical and conservative groups was the same, while the long-term function of the surgical group was better. A hypothesis proposed to explain this phenomenon in the long term is based on the inherent disadvantages of conservative treatment [19]. On the basis of the points mentioned above, it would seem that there are also many controversies on the optimal management of rotator cuff tears.

There are also many controversies among the choice of the optimal and most effective nonoperative managements including ice, simple exercises, medications, and progress to more intensive physical therapy, hyaluronic acid injections, CSI, biological augmentation with platelet-derived products (PRP), marrow stimulation, and so on [20]. Therefore, we found it essential to compare the efficacy of some of the most common conservative therapies including physiotherapy and CSI separately and simultaneously.

In the present study, despite the significant decrease in VAS and DASH scores in all three groups, no significant differences were observed between the two physiotherapy treatment approaches and CSI in the VAS and DASH scores. Instead, people who received both treatments showed significantly lower VAS as well as DASH scores than the other two groups. Regarding this issue, there are some studies that reported similar aforementioned results and recommended the combination of CSI and physical therapy in comparison with physical therapy or CSI alone [21, 22]. Burger et al. and Mehtap et al. also did not find any significant difference in the efficacy between physiotherapy and CSI, but they did not evaluate the influence of the simultaneous therapies [23, 24]. Hay et al., on the other hand, reported a significant better long-term outcome in the physiotherapy group in comparison with CSI even though he did not apply the combined therapies [25]. On the contrary, Koester et al. did not suggest the CSI in rotator cuff tears [26]. Therefore, it can be said that the combination therapy of CSI and physiotherapy is superior to each management alone for decreasing the pain and disability in rotator cuff tearing. Further studies are required regarding the exact dose and duration of corticosteroid therapy and finding the optimal technique of physiotherapy.

As to the ROM evaluation, in the present study, it was observed that therapies containing the steroid injection approach (steroid injection and steroids + physiotherapy) had a more dramatic effect on improving the patients' ROM than physiotherapy alone. Similar results were reported by Boudreault et al., reporting that corticosteroid injection had a significant effect on the range of motion of the shoulder in abduction. Conversely, Burger et al. and Mehtap et al. did not report a significant difference between the two treatment approaches, which is inconsistent with the present study. This may be due to the fact that in the Burger et al.'s study, the subjects were the patients with rotator cuff syndrome, and in the Mehtap et al.‘s study, the patients with shoulder pain were evaluated, but the present study was performed on patients with complete rotator cuff rupture. To sum up, everything that has been stated so far, the effect of combined treatment (corticosteroids + physiotherapy) can be far better than any of the treatments alone in the range of motion and performance of individuals.

Despite many positive considerations, this study had few limitations. No program was designed for long-term follow-up of patients. Furthermore, there was no control group to compare the effect of any interventions with no treatment. Likewise, although the patients were instructed not to use NSAIDs or herbal and home remedies either orally or systemically or topically during the study, we cannot be sure about it. Because we cannot follow the patients when they are out of the clinic.

## 5. Conclusions

Among the conservative approaches to complete rotator cuff rupture, a combination of steroid injection and physiotherapy can be more effective than either approach alone in both reducing pain and improving shoulder function and range of motions.

## Figures and Tables

**Table 1:** Gender frequency distribution of patients in the three intervention groups.

Variable	Frequency *n* (%)		*p* value
Physiotherapy	Female	27 (84.4)
	Male	5 (15.6)

Corticosteroid injection	Female	28 (87.5)
	Male	4 (12.55)

Physiotherapy + corticosteroid injection	Female	26 (81.3)	0.789
	Male	6 (18.8)	

Total	Female	81 (84.4)
	Male	15 (15.6)

**Table 2:** The effect of different management on pain-related scores and range of motions.

Variable	Physiotherapy			Corticosteroid injection			Physiotherapy + corticosteroid injection			*p* value	
	Before INT^a^	After INT	*p* value	Before INT	After INT	*p* value	Before INT	After INT	*p* value	Before INT	After INT
DASH^b^	57.21 ± 4.15	48.77 ± 4.0	0.000	57.29 ± 3.60	47.83 ± 3.52	0.000	56.77 ± 3.65	45.91 ± 4.12	0.000	0.797	0.013
VAS^c^	5.53 ± 0.62	4.00 ± 0.91	0.000	5.78 ± 0.70	4.18 ± 0.64	0.000	5.59 ± 0.66	3.56 ± 0.66	0.000	0.299	0.004
Flexion°	144.9 ± 9.7	164.9 ± 7.9	0.000	142.1 ± 10	169.6 ± 7.6	0.000	141.2 ± 9.2	170.6 ± 7	0.000	0.281	0.007
Extension°	61.9 ± 5.39	62.46 ± 7.79	0.406	59.4 ± 4.9	60.5 ± 4.43	0.000	60 ± 5.63	60.96 ± 5.63	0.029	0.140	0.290
Abduction°	119.1 ± 10.36	143.0 ± 8.97	0.000	116.34 ± 10.2	148.9 ± 8.8	0.000	118.7 ± 12.5	154.7 ± 9.8	0.000	0.555	0.000
Internal rotation°	36.6 ± 4.3	56.9 ± 3.2	0.000	33.81 ± 4.7	59.5 ± 3.2	0.000	35.4 ± 4.9	60.1 ± 4.12	0.000	0.060	0.001
External rotation°	50.4 ± 5.5	66.7 ± 4.2	0.000	48.6 ± 5.5	70.9 ± 4.3	0.000	49.0 ± 5.5	70.7 ± 3.8	0.000	0.236	0.000

^a^INT: intervention; ^b^DASH: disabilities of the arm, shoulder, and hand; and ^c^VAS: visual pain scale.

## Data Availability

SPSS data of the participant can be requested from the authors. Please write to the corresponding author if you are interested in such data.
